# Mortality and *Clostridium difficile* infection: a review

**DOI:** 10.1186/2047-2994-1-20

**Published:** 2012-05-30

**Authors:** Brett G Mitchell, Anne Gardner

**Affiliations:** 1 School of Nursing, Midwifery and Paramedicine, Australian Catholic University, Dickson, PO BOX 256, ACT, Australia; 2 Research Associate, National Centre for Clinical Outcomes Research (NaCCOR), Australian Catholic University, Sydney, Australia

**Keywords:** *Clostridium difficile* infection, *Clostridium difficile* associated diarrhoea, Mortality, Death

## Abstract

**Background:**

*Clostridium difficile* infection (CDI) is a common cause of diarrhoea in hospitalised patients. Around the world, the incidence and severity of CDI appears to be increasing, particularly in the northern hemisphere. The purpose of this integrative review was to investigate and describe mortality in hospitalised patients with CDI.

**Methods:**

A search of the literature between 1 January 2005 and 30 April 2011 focusing on mortality and CDI in hospitalised patients was conducted using electronic databases. Papers were reviewed and analysed individually and themes were combined using integrative methods.

**Results:**

All cause mortality at 30 days varied from 9% to 38%. Three studies report attributable mortality at 30 days, varying from 5.7% to 6.9%. In hospital mortality ranged from 8% to 37.2%

**Conclusion:**

All cause 30 day mortality appeared to be high, with 15 studies indicating a mortality of 15% or greater. Findings support the notion that CDI is a serious infection and measures to prevent and control CDI are needed. Future studies investigating the mortality of CDI in settings outside of Europe and North America are needed. Similarly, future studies should include data on patient co-morbidities.

## Background

The spectrum of diseases caused by *Clostridium difficile* ranges from uncomplicated diarrhoea to pseudomembranous colitis and toxic megacolon, and is often termed ‘*Clostridium difficile*-associated diarrhoea’ or ‘*Clostridium difficile* infection’ (CDI) [1-4]. Around the world, the incidence and severity of CDI has increased, particularly in the northern hemisphere [5]. This increase appears to be driven by a number of factors, including large outbreaks of CDI in hospitals, a change in circulating strains of *C.difficile*, and factors such as inappropriate antibiotic usage and poor standards of environmental cleanliness [6-10]. This paper presents a review of studies that have investigated mortality and CDI in hospitalised patients.

## Methods

### Design

An integrative review design was used. An integrative design was chosen because it summarises empirical and theoretical literature and allows for the synthesis of results when study designs and methodologies vary between studies. As a result, it provides a more comprehensive understanding of a particular issue [11].

### Search Methods

The literature was accessed through searches on two electronic databases—Medline and Pubmed—limited to the period 1 January 2005 to 30 April 2011. This limitation reflects the recently changing epidemiology of CDI. Other limits included only searching literature published in English, and studies involving humans. Key search words used were ‘*Clostridium difficile* and mortality’ and ‘*Clostridium difficile* and death’. These searches were combined, with duplicate studies removed.

The next step of the search strategy process involved a preliminary review of the articles. Only prospective or retrospective articles that were case controlled, cohort or reviews were included. Additionally, articles were only included if they examined the mortality of hospitalised patients and were not limited to CDI in a specific patient group, for example, a person with cancer. Only articles that did not use CDI as a comparison group were included, for example, mild versus severe CDI and related mortality. The rationale for excluding such articles was to gain a better understanding of CDI in the general hospitalised population rather than those with a specific illness.

A second review of the articles was undertaken, and this step excluded articles that did not examine mortality at fixed intervals, for example, 30 or 90 days. The inclusion of studies that documented mortality at fixed intervals was chosen in an attempt to assist the pooling of data during data analysis.

### Search Outcome

**Figure 1:**
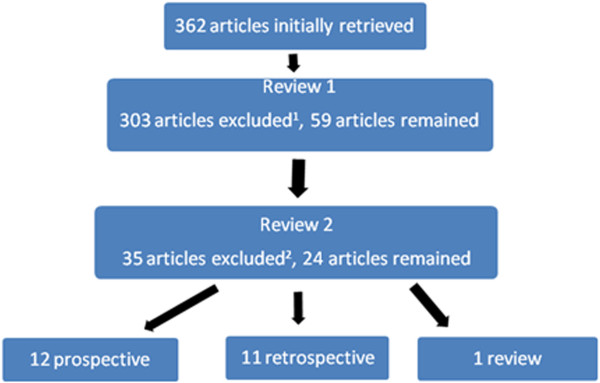
** Summary of search strategy. ***Notes.*^1^ Articles were excluded if they were not case controlled, cohort or reviews or did not examine mortality in hospitalised patients. ^2^ Articles were excluded if they did not examine mortality at fixed intervals.

The initial search yielded 362 articles. Following the first review, 303 articles were excluded and 59 articles remained. The second review, which excluded articles that did not examine CDI mortality at fixed intervals, resulted in a further exclusion of 23 articles. A total of 26 articles remained after the second review. Figure 1 provides a summary of the search strategy process.

### Quality Appraisal

All papers included in this review were published in peer-reviewed journals.

### Data abstraction and synthesis

All articles were analysed sequentially. The author, year, purpose, study design, data collection and analysis were reviewed. Data on mortality at fixed intervals were extracted from the studies and a table was populated (see Table 1).

**Table 1:** Summary of reported mortality from literature

**Author**	**Study type**	**Country**	**30-day mortality**	**Mortality at endpoint (other)**	**Participants**
Arvand, Hauri, Zaiss, Witte & Bettge-Weller (2009)	Prospective multicentre	Germany	25%	-	41
Bhangu, Bhangu, Nightingale & Michael (2010)	Cohort	UK	38%	-	158
Bishara, Peled, Pitlik & Samra (2008)	Case control	Israel	15% case	-	Total 217
			11.5% control (NS)		52 cases
Cadena et al. (2010)	Cohort	US	16%	29% at 90 days	129
Chung et al. (2010)	Cohort	Taiwan	23.3%	37.2% IHM	86
Cloud, Noddin, Pressman, Hu & Kelly (2009)	Cohort	US	-	12.1% IHM	272
Dubberke et al. (2008)	Case control	US	-	38% cases at 180 days	Cohort = 18,050 390 cases
				12% non-cases at 180 days	
Fenner et al. (2008)	Cohort	Switzerland	9%	-	78 cases
Gasperino et al. (2010)	Cohort	US	16% cases, 5% controls	20% cases IHM (cases)	216,108 cases
				8% IHM (controls)	
Gravel et al. (2009)	Prospective multicentre	Canada	16.3%	-	1430
			5.7% attributable		
Gulihar, Nixon, Jenkins & Taylor (2009)	Cohort	UK	19% (cases)	67% cases at 180 days	170 cases
			9% match cohort	29% matched cohort at 180 days	3,247 matches
Karas, Enoch & Aliyu (2010)	Review	UK	Not applicable	Not applicable	Not applicable
Kenneally et al. (2007)	Cohort	US	36.7%	-	278
			6.1% attributable		
Labbe et al. (2008)	Cohort	Canada	23.9%	-	230
Loo et al. (2005)	Prospective multicentre	Canada	24.8%	-	1703
			6.9% attributable		
Lyytikainen et al. (2009)	Cohort	Finland	-	14% at 30 days post-discharge	8 093
Marra, Edmond, Wenzel & Bearman (2007)	Cohort	US		17.2% at 14 days	58
				27.6% IHM	
McGowan et al. (2011)	Cohort	UK	35.5%	13.4% at 7 days	2,571
				20% at 14 days	
				58.7% at 1 year	
Musher et al. (2005)	Prospective observational	US	-	27% at 90 days	207
Pant et al. (2010)	Cohort	US	13.6%	-	184
Shears, Prtak & Duckworth (2010)	Cohort	UK	24.7%	30.0% at 90 days	227
Sundram et al. (2009)	Case control	UK	14.4% at 28 days (extrapolated)	11% at 3 days (027)	97 cases
				3% at 3 days (106)	97 controls
Wilson et al. (2010)	Cohort	UK	35.9%	-	128
Zilberberg, Shorr, Micek, Doherty & Kollef (2009)	Cohort. Secondary analysis of Kenneally et al. (2007)	US	26.9% <65 years	-	278
			45.2% >65		

Note. IHM = in-hospital mortality.

## Results

### Study Characteristics

Of the 24 articles included, 12 were prospective, 11 retrospective and one was a review. Eighteen studies reported mortality at 30 days or less, with 12 studies reporting mortality at further endpoints, predominantly 90 or 180 days. One study that reported 30-day mortality used 30-day post-discharge as the definition [12]. The choice of 30 days post-discharge as the method used to examine mortality was made due to limitations in available data, which should be noted when considering results from this study. Four studies examined in-hospital mortality in addition to 30-day mortality. The reported mortality in each of these studies is detailed in Table 1 and is explored in more detail later.

The search strategy used to identify articles for this review did not identify the same articles in the review published by Karas, Enoch & Aliyu (2010). Seven articles included in the review by Karas, Enoch and Aliyu [13] were not include in our review. These studies did not meet our inclusion criteria and were excluded if the participants were from a selected group. For example, they had a specific strain of CDI or had severe CDI. Conversely, our study did identify and included 14 studies not used by Karas, Enoch and Aliyu [13]. The primary reason for this discrepancy was the recent publications of studies, with 11 of these 14 published in the past two years. The review by Karas, Enoch and Aliyu [13], also focussed on attributable mortality.

### Reported mortality

As reported in the included studies, mortality varied considerably, both in the type of mortality calculated and the figures represented. The study design, exclusion criteria of participants and the data collected in the studies varied, making the pooling of data for analysis impossible. All-cause mortality at 30 days varied from 9% to 38%, as shown in Table 1. Three studies reported all-cause mortality at 90 days, with a range of 27% to 30% [14-16]. Similarly, three studies report attributable mortality at 30 days, varying from 5.7% to 6.9% [17-19]. In-hospital mortality was reported in four studies and ranged from 8% to 37% [20-23].

Eighteen studies documented all-cause mortality at 30 days, or 28 days in the case of the study undertaken by Sundram et al. [24], which examined two strains of *C.difficile* (027 and 106) and their respective mortality. The total cohort size was larger than the two groups combined; therefore, it was unclear whether participants with other strains of *C.difficile* died. Acknowledging the limitations of different study designs, and excluding the study undertaken by Sundram et al. [24], 2,041 of 7,774 persons (26.3%) died within 30 days of CDI diagnosis. These figures include people identified with CDI in these studies who died within 30 days (all-cause mortality) [14,15,17-20,22,25-34].

The manner in which mortality was analysed and presented in the studies differed; however, there were some similarities. In several studies, participants were divided into those with CDI and those without [19,22,27,35]. Other studies divided participants into two groups: survivors and non-survivors [18,26,33]. Regardless of the methods used, similar data analysis involved using mortality at a fixed period. Commonly, X^2^, or Fisher’s exact test, was used for categorical data analysis, while a *t* test was used for continuous variables [14,20]. Kaplan–Meier survival analysis and regression model were used in some studies [18,23,28,30,32,33]. Not all studies undertook adjustment for variables when determining mortality rates. Despite collecting basic demographic data on cases, Charlson co-morbidity data and exposure to recent medication such as antimicrobials, the study undertaken by Chung et al. [20] did not adjust for any variables in the calculation of hospital mortality and all-cause mortality.

In addition to studies comparing survivors and non-survivors, or those with and without CDI, some studies examined mortality by age group [17,31,34]. In the retrospective cohort study undertaken by McGowan et al. [31], age groups were divided into decades, with those under the age of 40 years grouped together. A significant difference in 30-day mortality was found for the three groups aged over 60 years, using <60 yeardsas the reference group (*X*^2^ = 65.82; df: 2; P < 0.05). The study did not examine the effect of co-morbidity on outcome [31]. Similar to the work of McGowan et al. [31], a prospective multi-centred study of 1,430 participants demonstrated that age-specific mortality increased sharply after 60 years of age [17]. These two studies demonstrate the importance of collecting and analysing age-related data.

Some studies presented data that compared groups based on the causative strain of *C.difficile*[24,30,31]. The retrospective cohort study undertaken by Labbe et al. [30] found that patients affected by *C.difficile* ribotype 027 were twice as likely to die within 30 days of diagnosis than patients infected with other ribotypes. Similar results were demonstrated in a prospective case-controlled study comparing persons with CDI caused by ribotype 027, 106 and all other ribotypes [24].

## Discussion

The majority of studies identified in this review were undertaken in the United States (nine), United Kingdom (seven) or Canada (three). No studies were included from an Asian or Asian Pacific setting. As there are different circulating strains of CDI in different countries, this is an important issue because mortality for persons with CDI in different regions may vary. A higher incidence of CDI compared to other countries with data on CDI incidence, coupled with an increased focus on CDI due to high-profile hospital outbreaks, may provide some insights into the number of included articles from these countries.

The majority of studies used the identification of toxins A or B via EIA or ELISA as part of the case definition of CDI. However, there were variations in which faecal samples were tested for *C.difficile*. The effect of such variations on reported mortality remains unknown. However, it would be logical to assume that a less sensitive laboratory detection approach may identify fewer cases in a given population. This has the potential to underestimate the incidence of CDI and, thus, the total number of people who died after having CDI. The argument for a standardised testing approach for CDI has previously been made [36].

A co-morbidity score, such as the Charlson co-morbidity index, was collected in a limited number of studies [18-21,23,30,33,35]. A number of studies used other tools such as the American Society of Anaesthesiologists (ASA) grade or Acute Physiology and Chronic Health Evaluation (APACHE) II, while a number of studies collected data on co-morbidities but did not specify how or why they chose those co-morbidities [14,17,24,27,28,34,37]). The admitting diagnosis was collected for two studies [19,23]. The variation in the use of co-morbidity data makes comparisons between studies challenging and potentially limits the validity of results. Where co-morbidities were collected, they were shown to influence mortality in patients with CDI [18,27]. In one instance where co-morbidity data were not collected, the researchers recognised this limitation and suggested that this area be included in future research [31].

The manner in which data were collected about drug therapy, surgery and nasogastric tube exposure were not reported consistently, with different periods and a range of procedures used. Further, in these studies, mortality was one outcome being examined alongside other variables such as risk factors for CDI. If a study was to examine CDI mortality as the single outcome, then the usefulness of including data on some of the data described above could be limited, particularly as there is no standardised method or definition used.

## Conclusion

There was limited homogeneity between the studies included in this review. Differences in study design, patient groups and data collected from participants were found. Some studies lacked demographic and co-morbidity data, which is a similar finding to the review undertaken by Karas, Enoch and Aliyu [13]. In general, all-cause 30-day mortality appeared to be high, with 15 studies indicating a mortality of 15% or more. Several studies demonstrated that increased age was a risk factor for mortality, indicating the need to include and analyse such data in future studies.

No articles from an Asian or Asian Pacific setting were included in this review. Even accepting all-cause mortality at the low end of the included studies in this review, CDI clearly have a significant effect on hospitalised patients. Studies investigating the mortality of CDI in settings outside of Europe and North America are needed so that the epidemiology of CDI in these regions can be understood.

Future studies examining the mortality of CDI should include basic demographic data, reporting of mortality at seven days, 30 days and 90 days after the first diagnosis of the CDI, the use of exclusion criteria and acquisition definitions as recommended in the international literature. In addition, the studies should use a co-morbidity index score such as the Charlson co-morbidity index. In doing so, pooling data becomes possible and comparisons between studies become easier. Similarly, the addition of data on antibiotic exposure will assist future meta-analysis on the role of antibiotics in CDI. The recommendations posed for future studies are similar to those described by Karas, Enoch and Aliyu [13] in the only review article identified. This review supports the notion that measures to prevent CDI are needed, given its impact on mortality.

## Competing interest

No conflict was declared by the authors for the study itself.

## Authors’ contributions

BM and AG were responsible for the study concept and design. BM performed data collection. BM and AG were responsible for data analysis and the draft of the manuscript. All authors have read and approved the manuscript.
